# Exploring Digital Twin-Based Fault Monitoring: Challenges and Opportunities

**DOI:** 10.3390/s23167087

**Published:** 2023-08-10

**Authors:** Jherson Bofill, Mideth Abisado, Jocelyn Villaverde, Gabriel Avelino Sampedro

**Affiliations:** 1 Research and Development Center, Philippine Coding Camp, Manila 1004, Philippines; jherson@philippinecoding.com; 2 College of Computing and Information Technologies, National University, Manila 1008, Philippines; mbabisado@national-u.edu.ph; 3 School of Electrical, Electronics and Computer Engineering, Mapúa University, Manila 1002, Philippines; jfvillaverde@mapua.edu.ph; 4 Faculty of Information and Communication Studies, University of the Philippines Open University, Laguna 4031, Philippines; 5 Center for Computational Imaging and Visual Innovations, De La Salle University, 2401 Taft Ave., Malate, Manila 1004, Philippines

**Keywords:** 3D printing, nozzle clogging, machine learning, smart monitoring

## Abstract

High efficiency and safety are critical factors in ensuring the optimal performance and reliability of systems and equipment across various industries. Fault monitoring (FM) techniques play a pivotal role in this regard by continuously monitoring system performance and identifying the presence of faults or abnormalities. However, traditional FM methods face limitations in fully capturing the complex interactions within a system and providing real-time monitoring capabilities. To overcome these challenges, Digital Twin (DT) technology has emerged as a promising solution to enhance existing FM practices. By creating a virtual replica or digital copy of a physical equipment or system, DT offers the potential to revolutionize fault monitoring approaches. This paper aims to explore and discuss the diverse range of predictive methods utilized in DT and their implementations in FM across industries. Furthermore, it will showcase successful implementations of DT in FM across a wide array of industries, including manufacturing, energy, transportation, and healthcare. The utilization of DT in FM enables a comprehensive understanding of system behavior and performance by leveraging real-time data, advanced analytics, and machine learning algorithms. By integrating physical and virtual components, DT facilitates the monitoring and prediction of faults, providing valuable insights into the system’s health and enabling proactive maintenance and decision making.

## 1. Introduction

Fault monitoring (FM) is a process that involves the detection and diagnosis of faults as soon as they occur caused by various events such as equipment faults, process faults, etc., through the continuous monitoring of a system [1]. This process is crucial to ensure the quality, efficiency, and safety of manufacturing processes, transportation, power plants, and other complex systems [2]. These complex systems have many interconnected components that are prone to malfunction or fail due to component degradation, which can lead to safety risks, costly downtime, a decrease in productivity, and other negative impacts [3]. The objective of FM is to detect issues promptly, enabling operators to carry out repairs and maintenance work proactively, before the system being monitored sustains damage or experiences downtime. Furthermore, its objective extends to provide more accurate information for the optimization of machine operation [4]. Analysis of device logs and system performance metrics, running diagnostic tests, and event correlation are examples of methods employed in FM operations. The method to be used in an operation will vary from system to system and what issues are most expected to occur.

Traditionally, FM involves using physical elements, including sensors, instrumentation, and manual inspection by machine operators responsible for collecting and analyzing data [5]. These elements can provide relevant data about the system’s behavior and performance, but they have several limitations. For instance, they may need help capturing the subtle and complex interactions of the components in the system; they may not provide real-time monitoring and analysis; and they may be resource-intensive and costly to implement and maintain. To overcome these challenges, advanced fault monitoring systems, including but not limited to condition-based monitoring [6], predictive maintenance [7], prognostics and health management [8], and Internet-of-Things-based monitoring [9], have been developed that can automatically detect and diagnose faults in complex systems. These monitoring systems utilize combinations of various technologies such as data analytics, artificial intelligence, computer vision, control systems, etc, for the pattern identification that indicates the presence of a fault in real-time and massive data set analysis, which ensures a high level of precision and accuracy in capturing the interactions between components of the system and thus has an edge over traditional FM approaches. Currently, the use of digital twins is emerging as a promising technology for FM.

Digital Twins are digital counterparts of physical entities such as equipment and systems supplied with real-time data that can span from its atomic to geometric level, enabling a more holistic approach to understanding and optimizing the performance of these physical entities [10]. Digital Twin (DT) aims to integrate concepts of simulation, twinning, data analytics, optimization, and monitoring into a single technology. The objective of DT technology is to perform simulations on the virtual counterpart in the same manner as tests will be conducted on the physical counterpart, which can provide a comprehensive and detailed view of the system’s behavior and performance that can help identify the subtle faults that traditional monitoring methods may miss [11]. For instance, DTs can continuously collect real-time information on the temperature, pressure, vibration, and other metrics of a component or system through various sensors, and it can be analyzed to detect and diagnose faults that can occur and consequently facilitate corrective action before failure [12]. This can help minimize the risk of complete system failure and costly repairs, and it can reduce the downtime experienced due to equipment maintenance. This can be particularly useful in cases where the physical entity is located remotely or is difficult to access or where real-time monitoring could be more practical [13]. In spite of these advantages of using DT technology for FM, there are still challenges to be overcome, and consequently, there is an opportunity to improve this technology in this application.

In using DT technology for FM, accurate model construction in terms of the technical characteristics, complex processes, performance when subjected to interference, etc. is highly vital to make it as parallel as possible to its physical counterpart [14]. In this necessity arises one of the current challenges of DT technology for FM, as it requires extensive data and knowledge of the physical device/system which can be tedious and tremendously complex. Another challenge is the regular data collection requirement from sensors present in the system and the need to continuously update and maintain the DT’s model and algorithms as the system continuously changes over time, which can be resource-intensive. Finally, the data security concern in DT technology, as it involves accumulating and processing sensitive data from the systems, can be daunting; thus, organizations must have the right tools, processes, and personnel to manage their Digital Twin environments effectively.

Thus, this paper presents an overview of DT architecture, approaches, and data monitoring types for DT in FM and aims to summarize and synthesize the essential findings and contributions of the research of this technology (DT) in FM applications to identify the main themes, trends and highlight promising directions for future research. However, it must be noted that the discussed strengthes and weaknesses of each algorithm used for DT in FM are irrelative to each other due to their different applications. A brief explanation of FM and the current challenges and opportunities for improvement of DT in FM will be provided.

## 2. Methodology

A comprehensive review of the research work within the last five years on DT technology for FM was gathered from online databases, including Mendeley and Google Scholar. The papers were searched using keywords such as “digital twin”, “fault monitoring”, “fault diagnosis” and “condition monitoring”. Only journal articles and conference papers related to DT in FM were selected; review articles concerning the overview of DT technologies were excluded during the filtering process. Table 1 contains the list of DT in FM papers categorized by publication years. The majority of the research papers within the point of interest were published in 2021, followed by 2022.

This study only looked at publications from 2018 to 2022. Table 1 indicates that only two relevant results were obtained for 2018, while there were four for 2019 and six for 2020. Therefore, it can be inferred that 2019 and 2020 are the years when the research interest in Digital Twin-based fault monitoring started to pick up. Among the publication years considered in this survey, 2022 and 2021 have the highest publication count, with 17 and 30 publications, respectively. It shows from the data that DT-based FM gained traction in 2021, and 2018 is the approximate starting point of research in the field. The decrease in published work in 2022 could indicate that the topic has already been explored to a significant extent and there are less findings or novel ideas to be reported.

This paper provides a brief overview of DT technology and its potential applications in FM. The methodology used for our literature review, including the databases and search terms, is then described. The main body of the paper presents the key findings of the research articles reviewed, organizing them into themes and trends. Finally, the paper concludes by summarizing the key findings and discussing implications to future research.

## 3. Digital Twin Architecture

DT technology has gained considerable interest in recent years as a promising approach for fault monitoring in various industries, including manufacturing, transportation, and energy. A DT is an ultrarealistic digital model that mirrors the dynamic and static attributes from data of the physical asset that are always in sync with one another, wherein simulations can be run with the DT to examine the performance of the physical asset [74]. Furthermore, to consider a digital model to be a DT, it needs to be designed with three attributes: (1) the ability to synchronize with the real-time functionality of its physical equivalent, (2) the capability to perform simulations, and (3) active data acquisition. Once these three attributes are met, and the DT is integrated with artificial intelligence, it offers an autonomous system that allows the continuous simulation of alternatives that will improve predictive maintenance, process optimization, etc. The DT is transmitted and executed on cyberspace (cloud resource); hence, even if the connection is momentarily lost with the physical asset (device, system, component, etc.), the previously known status will still be known [75]. Since the DT can be stored in the cloud, it also provides the functionality of sharing the structured model in a global context.

### 3.1. Layers of Digital Twin Architecture

**Figure 1:**
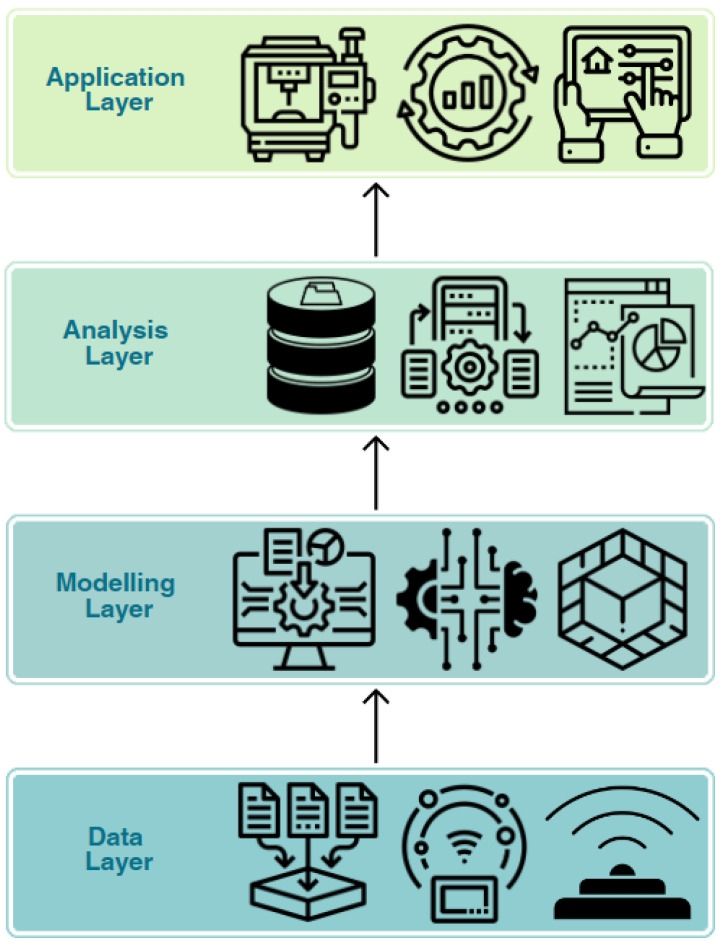
Four layers of DT.

**Figure 2:**
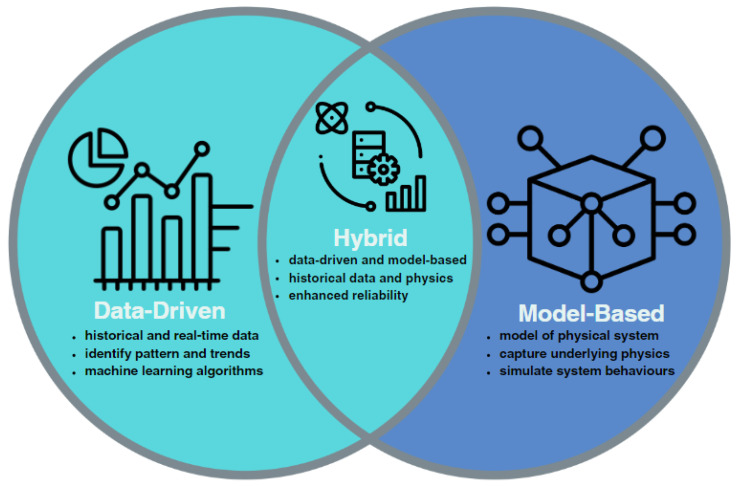
DT prediction methods.

A study on the integration of DT technology to a shop floor conveyor into the manufacturing control system by [76] presented that DT architecture, as seen in Figure 1 and Figure 2, comprises the data, modeling, analysis, and application layers. The (i) data layer is responsible for collecting and storing data from various sources, such as sensors, Internet of Things (IoT) devices, and other systems that generate data. These included various sensors installed on the conveyor to collect data on the process parameters, such as speed, temperature, and vibration. Next, the (ii) modeling layer is the central area where the model creation and calibration occurs, where a virtual model of a physical counterpart or process is created using various modeling techniques, such as mathematical modeling, 3D modeling, or machine learning algorithms, and it is calibrated using the data collected by the sensors and validated using the actual measurements obtained from the system. In the work of [76], a conveyor system that has various components, such as motors, belts, rollers, and sensors, and their interconnections, was made a dynamic model in the modeling layer. Then, the data collected are transmitted, processed, and analyzed from both the data layer and modeling layer to the (iii) analysis layer where information is extracted by means of various data analysis techniques (machine learning, statistical analysis, data mining, etc.) to identify patterns, correlations, or anomalies in the data. This layer covers activities that improve the performance and efficiency of a system through predictive maintenance, process optimization, or quality control. Lastly, the (iv) application layer provides interfaces and applications for users to interact with the digital twin, such as monitoring, control, or optimization. These included a control algorithm that optimizes the conveyor system based on the virtual model and corresponding real-time data as well as a user interface that enables operators to visualize the conveyor system, monitor the process parameters, and adjust the control settings based on the presented DT technology integration by [76].

### 3.2. Digital Twin Prediction Methods

The general essence of DT is to detect any abnormal conditions in the physical asset before it reaches a malfunction or failure, as all physical assets inevitably degrade over time, thus preventing various consequences (financial, environmental, and workforce safety). To ensure the reliability of the physical asset, monitoring the results of predictive simulation from the initial stage of degradation will serve as a basis for subsequent maintenance [77]. According to [78], there exist three types of prediction methods: data-driven, model-based, and hybrid, which combine the two. Data-driven methods depend on either historical data alone and identify matching patterns or both real-time data and historical data to estimate the future operating performance of a physical asset [79]. Furthermore, an advantage of the data-driven method is that it does not require generating all technical information regarding the equipment/system, it only requires data from the many sensors to be analyzed, and the data structure would wholly depend on the user [80]. Artificial intelligence methods, statistical methods, and reliability functions are some methods utilized in the data-driven approach. For example, Ref. [45] proposed a Data-driven DT Fault Diagnosis (DDFD) learning group that evaluates the operational conditions of machining tools used in automotive applications through deep transfer learning. To sense the temperature of the sampling tool, a k-type thermocouple is integrated with a cloud data acquisition system over a WiFi module. The DDFD approach achieved 92.33% accuracy, which is better than virtual [81] and physical [82] deep neural network models, which attained accuracies of 90.13% and 90.13%, respectively. Ref. [39] provides a data-driven approach to Smart Prognostics and Health Management (SPHM) of specifically a milling machine, using large amounts of data generated from shop floor devices for detecting the presence of a fault and estimating the Remaining Useful Life (RUL), and it highlights the need for a multi-faceted approach or framework with Prognostics and Health Management (PHM) which includes three phases: Setup and Data Acquisition, Data Preparation and Analysis, and SPHM Modeling and Evaluation. These three phases explain that predictive maintenance is a collection of methods (machine learning, deep learning, reliability, etc.). The SPHM framework’s effectiveness was proven in its fault detection and RUL estimation capabilities. As the data-driven method is heavily reliant on the operation data obtained by numerous sensors (a single sensor cannot detect all desired information) installed in a system, a drawback of this method arises regarding how sensors sometimes cannot be installed in specific areas or components of interest in the system, which makes data acquisition difficult and hinders the creation of a holistic representation of the physical asset [83]. The model-based method, on the other hand, relies on mathematical models of a physical system that simulate its behavior that can be derived from first principles or can be developed using data-driven techniques [84], and have its model parameters updated from measured data [85]. Furthermore, the model from this method reflects the performance of a system, with degradation dependent on its internal working mechanism, and it represents all links between various components within the system [80]. From this, the trend in performance degradation can be predicted. Ref. [51] presented a model-based simulation through the 3D finite element method known as the computational modeling technique, using parameters of both healthy and broken induction motors and motor current signature analysis to determine the impact of fault presence within the motors. The outcomes were evaluated in both time and frequency domains, and an artificial neural network was employed to categorize the current health of the motor model. The authors suggested the possibility of creating a parameterized database of healthy and faulty motors, which could be used to train fault diagnosis (FD) systems. According to [65], the model-based DT approach for FD can be a robust and cost-effective method that ensures the dependability and fault tolerance of systems, specifically in photovoltaic (PV) systems that use a mathematical analysis, simulation study, and experimental validation. Their approach allows the real-time estimation of the outputs characteristic to a PV energy conversion unit (PVECU) and diagnoses faults by generating and evaluating a residual error vector, the difference between the estimated and measured outputs, which showed that the proposed approach is capable of detecting the presence of a fault and classifying the type of fault existing in the PVECU, with fault detection and identification times ranging from less than 290 (micro s) to less than 1.2 s. This methodology illustrates greater fault sensitivity compared to existing approaches. The model-based method offers the users the freedom to simulate various scenarios with the system’s operation achieved through a myriad of data sheets and information with individual components in the system. A drawback of this method is its complexity and the need for technical experts to design and generate the model as accurately as possible [80]. Both approaches have their respective advantages and disadvantages, which are often case-specific. Hence, a third method fuses the two mentioned methods and adopts their advantages. The hybrid method combines first-principle and operation data. Based on [86], this method can be divided into three parts: data input, mechanism analysis, and data fitting. The mechanism analysis is deemed the most critical aspect of this approach, since it embodies the operation of the model. The performance information is taken from the operation data (data-driven) and analyzed through a first-principle mechanism (model-based) before the hybrid modeling. Ref. [52] employed this approach to propose a solution that tackles the issue of intelligent instrument FD, which has gaining prominence in the field of manufacturing. The suggested system comprises three layers: the data layer, control layer, and output layer. The data layer employs Micro-Electro-Mechanical Systems (MEMS) sensors and a Zigbee wireless transmission network to build a data connection between the physical endpoint and the virtual model. Their designed FD and prediction system for the indentation tested yielded an accuracy of 90%. These three methods of prediction have their own merits; the method most appropriate for a prognosis would depend on what the demands of the user would be.

## 4. Digital Twin in Fault Monitoring

The following literature review pertains to selected papers from different industries. It aims to outline the DT application in FM on equipment and system levels based on their relevance and contributions to the topic. Table 2 shows the summarized details on the application, approach, and performance of the papers discussed in Section 4.1, which have been chosen based on their completeness, reproducibility, and whether the information stated is redundant.

### 4.1. Equipment-Level Application

One of the main applications and a driver for the Industry 4.0 revolution is the PHM systems of various industrial components/equipment [87]. The equipment-level application of DT in different industries will be discussed from here. Ref. [49] discusses a novel implementation method that combines DT with the FD of large industrial equipment focused on rolling bearings. The proposed framework was evaluated by comparing real-world and simulated fault signals of the rolling bearing, giving information on the performance of the inner and outer race, using a sampling frequency of 12 kHz and 1750 rpm test speed. Pearson, Spearman, and Kendall’s statistical methods compared the signals yielding a *p*-value < 0.05, indicating that the simulation signal significantly matches the real-world signal. But as this is only a designed test, it may not fully amount to the bearing in the real-world scenario. Meanwhile, the study of [22] not only developed a technique for bearings but also concentrated on diagnosing crack types and sizes. This was achieved by utilizing a combination of a strict-feedback backstepping DT and a machine learning algorithm. This approach involved designing a DT that will model and estimate acoustic emission signals, generate an acoustic emission residual signal, and use a support vector machine to classify the crack type and size. To test the efficiency of the proposed technique, the bearing dataset from the Ulsan Industrial Artificial Intelligence Laboratory was used, resulting in an average accuracy of 97.13% and 96.9% for crack type diagnosis and crack size diagnosis, respectively. This approach shows a superior crack size and fault pattern identification; however, the inner-ball fault identification performance indicates that a more complex algorithm is needed for improved accuracy. For aviation, there is a significant difficulty in observing the status of an aircraft engine using traditional diagnosis methods. To aid in this difficulty, Ref. [18] proposes a hybrid FD method for the turbofan engine TFE-731. The status can be monitored online, featuring its high suitability for on-time monitoring. The proposed method employs Fast Orthogonal Search (FOS) due to its superiority in time-frequency analysis and spectral estimation compared to the fast Fourier transform due to FOS’s capability for finding orthogonal bases. In addition, the autoregressive moving average (ARMA) model is widely used for the system output prediction of DTs and can predict the RUL of a valve of an aircraft engine, but it can have difficulties with conforming with underlying system dynamics and capturing nonlinear relationships within the system. Practical implementation of the FOS-based ARMA data-driven approach results in a lower root mean square error and higher %variance accounted for (%VAF) of the FOS model compared with the results of the original model. This improvement validates its real-time effectiveness, showing high prediction accuracy and stability, suggesting that the method can be utilized in examining the irregular parts of an aircraft engine. In contrast, Ref. [17] focuses on the sensor diagnostics and PHM of the Wärtsilä 9L50DF marine dual fuel engine. The paper presents an Engine Diagnostics System (EDS) that enables intelligent engine monitoring, advanced sensor fault detection, and timely corrective actions. The proposed Unified Digital System (UDS) integrates the EDS with the diagnostic and control system of the engine, combining a data-driven model with functional and thermodynamic approaches to comprehensively diagnose sensor abnormalities, faults, and failures. While the failure and degradation of the mechanical engine components were not considered, simulation results show the UDS concept’s effectiveness, showing that it can capture engine sensor irregularities and restore engine operations to their original state by applying appropriate corrective actions. Ref. [33] study the DT of a switchgear cabinet, which is a piece of leading equipment for substations. Their methodology uses the random forest algorithm to construct a partial discharge pattern recognition model. The measured partial discharge patterns from the switchgear cabinet correspond to different faults: tip discharge, internal insulation discharge, particle discharge, and suspension discharge. Then, noise and random function-generated discharge pulse points are added to the measured data to create the test set. The random forest-based partial discharge pattern recognition model yielded an accuracy of 97% for the test set. A DT-assisted FD framework of railway point machines (RPMs), an essential component in railways that signals the movement direction of the train and simultaneously changes it, is discussed in the work of [34]. This work aims to identify the possible root causes for malfunctions of the RPM by measuring the current curve of its physical entity and comparing it with the current curve derived from its virtual counterpart. The strength of this approach is that it can realize the early detection of faults by using an improved curve thresholding method as opposed to existing classification methods. For automotives, centered on Li-ion battery packs of electric vehicles, Ref. [57] used a holistic data pipeline to transfer information such as the State of Charge (SOC) and State of Health (SOH) by reverse-engineering the diagnostics interface of a 2014 e-Golf to query for Unified Diagnostic Service (UDS) messages containing both battery pack and cell-individual data. An On-Board Diagnosis (OBD) logger was used to record the data with edge-processing capability, which was pushed into the cloud twin system using IoT technology. Battery models were fitted to the data, and cell individual internal resistance was inferred. State estimation of the battery was presented, and the data were coherent and relatively comparable to those from the literature. However, the confidence interval (CI) only amounted to 50%, and there is also a need for improvement in the reverse engineering of the battery as some of the internal resistance results had high variance requiring large sample amounts to establish statistically precise results.

### 4.2. System-Level Application

In the domain of system-level applications, Ref. [26] provides a framework for integrating multiple data sources and modeling techniques to enable the accurate diagnosis and prognosis of equipment faults in a factory setting. The networked proximity sensors collect real-time data from the factory floor. At the same time, maintenance log data are used to construct a high-fidelity simulation of each piece of equipment using a data-tagging technique. This simulation is then used to determine fault relationships and train a DT model, which is integrated into a detailed DES model of the factory. The co-simulation approach is demonstrated in a case study of a manufacturing line, showing that it can accurately predict equipment failures and optimize maintenance schedules to reduce downtime and improve productivity. This approach trains Bayesian networks (BNs) as a suitable machine learning (ML) algorithm to represent multilevel faults of complex hierarchical systems. Furthermore, BN allows the determination of how faults spread within and between different categories of faults. A single model can function for prediction and diagnostic purposes without requiring additional training, and the model can also account for the related uncertainties. The implementation of the Structural Intervention Algorithm is also discussed, which enables the detection of all possible directed edges and the distinction between a parent and an ancestor node of the BN. To avoid damaging the equipment, only noninvasive faults were examined in the testbed; however, the same approach can possibly be used for invasive faults. The validation experiments determined the Structural Hamming Distance (SHD) metric, using the “time to failure” as its parameter. Reduction of this parameter results in an enhanced BN structure, while increasing it does the opposite. This shows that the accuracy of the proposed approach is superior to traditional methods. However, the effectiveness of this approach is limited to the functionalities of the simulation software provided by the manufacturer. Ref. [20] focused on the improvement of operation efficiency of micro-grids by developing a smart framework based on cloud-edge integration used for the diagnosis of a micro-grid composed of three sub-micro-grids with different photovoltaic arrays under different operating conditions. The developed intelligent framework consists of three stages: Firstly, a micro-grid DT model was created on the CloudPSS platform using a DT approach. Secondly, a neural network-based FD model was developed by training it on the cloud server using data from the DT model. Finally, the trained model is downloaded to the edge device to conduct the offline FD of the microgrid, reducing the cloud server’s computational load. This is particularly important for maintaining the security and reliability of data storage and transmission. The neural network uses a Rectified Linear Unit activation function, and as the number of iterations increased, the loss value of the model decreased, resulting in a 95% accuracy. While the proposed method of DT using a cloud server for the model is economical, can execute real-time tasks for fault diagnosis, and has a relatively easy operation, it is possible that it cannot be applied with DT of other systems due to the difference in data dimensionality. Ref. [16] discussed a model-based DT FD system level of thermal–hydraulic high-pressure feedwater systems in nuclear power plants. The model-based approach makes it possible for virtual sensors to be utilized in the multi-component system and be placed in areas that would not be otherwise possible due to physical domain sensor restraints, which results in improved FD capabilities and automated monitoring of the DT model. The model construction begins with the decomposition of the system into its operational building blocks with functionalities that the first principles can explain. Measurements taken by the physical sensors are used to create the model, in hand with the virtual sensors used to determine the unmeasured process variables, relying on a combination of conservation laws and relations of components present in the system. After the measurements have been identified from both sensors, these will be used for the model calibration. The result of their model validation showed that their approach was able to indicate a recurring abnormality during the startup of the tested feedwater component, as confirmed by the plant operator, which says that although the model is functional, it is still reliant on human intervention. The work of [42] provides a simple discussion on the intelligent monitoring and maintenance of power grid substations using DT. The current approach for monitoring substations often presents complexities due to the independence of equipment and system stages from their design to operation and maintenance. This results in difficulty in creating a complete holistic life cycle of the substation. DT application will provide improved operation monitoring by reconstructing both the substation and its environment; then, the reconstructed model is fused with the mapping of the physical substation and thus enables a two-way coordinated interaction, real-time monitoring, FD, and security event warning. A DT estimator is initially used in the DT approach of [65] for the FD of distributed photovoltaic (PV) systems. The DT estimator can be either of the three DT prediction methods, and it analytically measures the real-time characteristics of a system and creates a digital emulation. The unique advantages of PV systems (high energy yield, scalability, performance reliability) are desirable in architecture. However, these systems are prone to a myriad of faults that degrade performance due to their complex outdoor installations, aging, and equipment weathering. Moreover, the faults occurring at PV energy conversion units (PVECUs) are difficult to discover and repair, as the PVECUs are numerous in each system. Hence, a robust DT approach for FD and Fault Identification (FI) uses the measured characteristics of the PVECU and compares it with the physical component to generate an error residual vector. The generated vector is analytically evaluated to diagnose the presence of a fault and its PVECU component (PV Panel, Power Converter, Electrical Sensor) origin. Experimental results demonstrate that the approach performs well in realistic outdoor conditions, can recognize real-time faults, and precisely classifies their origin with relatively higher sensitivity and robustness (yielding an FI window = 2 ms) than existing approaches. However, it was noted in their paper that there is a trade-off between the sensitivity and robustness in this approach, meaning that a higher sensitivity can make it prone to false alarms. In [32], a DT-enabled fault detection and diagnosis process is used for a building’s Heating, Ventilation, Air Conditioning (HVAC) system. As the excess use of HVAC sensory data degrades the performance of the DT, a Bag-of-Words (BoW)-based feature extraction and selection method is used to detect data and determine whether it is normal or faulty data, and it will be then appropriately tagged. A brick schema, which contains the description and intricate relationship of physical, virtual, and logical assets existing in the construction industry, is validated as the ontology to set up the semantic model for tagging faulty data. Sets of sensors were deployed in different zones of the HVAC system of the research facility of Oak Ridge National Laboratory; the data from these sensors correspond to five specific fault types (fault a − fault e): excessive filtration, +4 °F and −4 °F at zone 103, +4 °F and −4 °F at zone 205 the system. The resulting True Positive Rates (TPRs) are calculated from an adopted goodness-of-fit test that determines whether faults can be detected and can be seen in Table 2. Using the Brick schema as the basis for tagging normal and faulty data allows a better representation of the physical components and their complex functionalities, resulting in the flexible development of smart buildings. The difficulty arises when the application and corresponding ontology required is of a mechanism/system unavailable to Brick.

While the implementation of DT in FM presents unique benefits, several challenges need to be addressed to refine the application of this technology. One main challenge is the need for more standardization and interoperability among different equipment and systems, leading to difficulties when integrating existing monitoring systems with proposed DT models [20]. This results in a lack of scalability, limited data sharing, and increased costs due to customized solutions across different systems. As DT technology relies heavily on data used for DT model calibration, the accuracy and reliability of data are vital, which can be affected by factors such as the consistency and completeness of the data and the quality of the sensors. This requires precise selection, calibration, sensor placement, and data pre-processing techniques. An interesting aid to this problem would be the approach presented in [16], using virtual sensors to secure the completeness of data. Tremendous amounts of data are required for the model creation, which can result in privacy and security concerns. It is critical to guarantee the data’s confidentiality, integrity, and quality for the successful implementation of DT in FM [31]. Significant computational resources are essential, which can be an issue for systems with constrained resources; therefore, it is necessary to continuously develop efficient and scalable algorithms. The development of accurate and reliable DT models requires a deep understanding of the underlying processes of equipment or a system wherein the lack of domain knowledge and expertise presents itself as a significant challenge.

Implementing DT in FM (and consequently FI and FD) can have several benefits. One of these is the ability to improve from the current reactive maintenance approach to a more proactive predictive maintenance approach. Maintenance activities can be carried out before a failure occurs, promoting increased equipment/system uptime and significant cost savings by performing maintenance activities. These are improved through the real-time monitoring and prediction of the equipment/system’s imminent failure. Furthermore, DT in FM enables the development of more precise and reliable prognostic models that will greatly facilitate the optimization of equipment design and bring greater efficiency and increased product quality. It can also allow the data integration of different equipment and systems to obtain a more comprehensive view of the entire system, establishing better monitoring and optimization at the system level. To increase revenue streams and improve customer satisfaction, developing new business models that provide real-time information about the equipment/system and conduct predictive maintenance can be explored.

## 5. Conclusions

This paper presents an overview of DT technology and the factors explaining why this technology has emerged as a promising approach for fault monitoring in various industries, such as the potential for improved predictive maintenance, process optimization, and quality control. Furthermore, when integrated with artificial intelligence, DTs can provide autonomous systems capable of simulating alternative scenarios and enhancing the reliability and performance of physical assets. Case studies from various industries demonstrate the effectiveness of DTs in fault monitoring. At the equipment level, DTs have been used to monitor rolling bearings, detect crack types and sizes, diagnose faults in aviation turbofan engines, and enable sensor diagnostics in marine engines. System-level applications integrate multiple data sources and modeling techniques to accurately diagnose and accurately predict equipment faults in manufacturing environments. Despite the benefits offered by DTs, challenges exist. Data-driven methods rely heavily on sensor data availability and can be limited by the difficulty of installing sensors in specific areas or components. Model-based methods require accurate modeling and calibration, demanding technical expertise and effort. Hybrid methods aim to address these challenges by combining the strengths of data-driven and model-based approaches.

In conclusion, DT technology holds great promise for fault monitoring in various industries. Its integration with artificial intelligence enables the development of autonomous systems that continuously simulate and optimize the performance of physical assets. However, carefully considering the appropriate prediction method and addressing challenges related to data acquisition and model accuracy are essential for successful implementation. DTs’ continued advancement and application are expected to significantly improve fault monitoring, predictive maintenance, and overall operational efficiency in the industrial sector. Furthermore, exploring the use of blockchain and decentralized networks can possibly address issues with data privacy and security concerns.

## Figures and Tables

**Table 1:** List of Digital Twin applied to fault monitoring papers.

Year of Publication	DT in FM	Publications
2022	18	[15,16,17,18,19,20,21,22,23,24,25,26,27,28,29,30,31,32]
2021	32	[33,34,35,36,37,38,39,40,41,42,43,44,45,46,47,48,49,50,51,52,53,54,55,56,57,58,59,60,61,60,61]
2020	6	[62,63,64,65,66,67]
2019	4	[68,69,70,71]
2018	2	[72,73]
Total	59	

**Table 2:** Summary of DT in FM.

PY	Ref.	Scope	Industry	Application	Prediction Method	Proposed Algorithm	Performance
2021	[49]	Equipment	Manufacturing	Deep Groove Ball Bearing	Data-Driven	Detail Parameter	r = 0.79, *p* < 0.05
2021	[22]	Equipment	Industrial	Cylindrical Rolling Bearing	Hybrid	Strict Feedback DT and ML	Acc: 97.13%
2021	[18]	Equipment	Aviation	Turbofan Engine	Hybrid	FOS-Based ARMA	%VAF = 99.9%
2021	[33]	Equipment	Energy	Switchgear Cabinet	Data-Driven	Random Forest Algorithm	Acc: 97%
2021	[34]	Equipment	Transportation	Railway Point Machine	Data-Driven	Current Curve Diagnosis	N/A
2022	[17]	Equipment	Maritime	Diesel Engine	Data-Driven	Unified Digital System	%Error = 1.1%
2022	[57]	Equipment	Automotive	Battery Packs	Hybrid	OBD Data to Cloud-Based DT	CI = 50%
2021	[26]	System	Manufacturing	Assembly Line Robots	Data-Driven	Structural Intervention	SHD Score = 9
2021	[20]	System	Energy	Microgrid	Data-Driven	Connected Neural Networks	Acc: 95%
2021	[16]	System	Nuclear	High-Pressure Feedwater System	Model-Based	Mass Balanced Virtual Sensors	N/A
2022	[42]	System	Energy	Power-Grid Equipment	Hybrid	N/A	N/A
2022	[65]	System	Energy	Smart Building	Model-Based	Prototype Validation	Small FI Window = 2 ms
2022	[32]	System	Construction	Smart Building	Hybrid	BoW-Based Feature Extraction and Selection	TPRs: fault a = 63.8% fault b = 61.4% fault c = 53.9% fault d = 68.7% fault e = 70.2%

## Data Availability

Not applicable.

## Abbreviations

The following abbreviations are used in this manuscript:

FM	Fault Monitoring
DT	Digital Twin
IoT	Internet of Things
ML	Machine Learning
RUL	Remaining Useful Life
SPHM	Smart Prognostics and Health Management
PHM	Prognostics and Health Management
FD	Fault Diagnosis
PV	Photovoltaic
MEMS	Micro-Electro-Mechanical Systems
UDS	Unified Digital System
SOC	State of Charge
SOH	State of Health
OBD	On-Board Diagnosis
DES	Discrete Event Simulation
BN	Bayesian Network
SHD	Structural Hamming Distance metric
PVECU	PV Energy Conversion Unit
FI	Fault Identification
